# Prevalence and Predictors of Hypocalcaemia among Adolescent Girls in Rural Public Secondary Schools in South-South Nigeria

**DOI:** 10.4314/ejhs.v31i6.13

**Published:** 2021-11

**Authors:** Iyabo Oyibo, Patrick Gold Oyibo, Blessing Osie-Efetie

**Affiliations:** 1 Department of Paediatrics, Princess of Wales Hospital, Bridgend, UK; 2 Department of Community Medicine, Faculty of Clinical Sciences, College of Health Sciences, Delta State University, Abraka, Nigeria; 3 Department of Health and Safety Education, Delta State University, Abraka, Nigeria

**Keywords:** Hypocalcaemia, prevalence, predictors, adolescent girls, south-south Nigeria

## Abstract

**Background:**

Adolescent girls are at risk of developing skeletal inadequacy due to an imbalance between calcium intake and high requirements of calcium during this period of increased modeling and skeletal consolidation. This study assessed the prevalence and predictors of hypocalcaemia among adolescent girls in rural public secondary schools in south-south Nigeria.

**Methods:**

This was a cross-sectional study conducted to assess the prevalence and predictors of hypocalcaemia among 238 adolescent girls selected by a multi-stage sampling technique. Data was collected using a semi-structured questionnaire which was interviewer-administered. Descriptive and inferential analysis of data collected was carried out using the IBM SPSS version 22 software.

**Results:**

Over half (53.3%) and 75.2% of the participants were in their late adolescence period (17–19 years) and belonged to the lower social class level. Over one-quarter (30.7%) of the participants had hypocalcaemia. Participants who were in their mid-adolescence period (14–16 years) (OR= 2.38; 95% CI: 1.23–4.57), who skipped lunch (OR= 2.92; 95% CI: 1.35–6.34), who skipped breakfast (OR= 3.60; 95% CI: 1.65–7.83) and were in senior secondary 1 class (OR= 4.76; 95% CI: 1.21–18.75) had 2, 3, 4, and 5 times higher likelihood respectively of having hypocalcaemia. Participants who consume milk daily, who consume milk weekly and who had normal weight had 81.0%, 60.0% and 72.0% decreased odds respectively of having hypocalcaemia.

**Conclusion:**

The study brings to the fore a high prevalence of hypocalcaemia among the participants. Educational interventions targeted at parents to support adolescent girls to take calcium-supplements and calcium-rich meals should be implemented.

## Introduction

Adolescents are persons between the age 10–19 years (1,2). The adolescence period is a developmental period of transition that occurs in every human being from childhood to adulthood (1,2). It is a period of dynamic changes characterized by accelerated physical, biological, biochemical, cognitive and emotional development (3). These changes are closely related to their nutritional status. Dietary calcium has been identified as of one the nutrients of great concern for adolescents. Calcium is an important element for maintaining bone mineral homeostasis (4,5). Adequate calcium intake promotes better bone balance and with a better bone balance, the risk of osteoporosis is lowered (6). Inadequate calcium intake in childhood and adolescence might alter peak bone mass with adverse implications later in life (7). Among the nutritive factors, calcium seems to be the most important determinant of peak bone mass in adolescents (7). Therefore, calcium deficiency or factors that interfere with calcium uptake may be critical at this period of life.

Many female adolescents are at risk of developing skeletal inadequacy due to an imbalance between calcium intake and high requirements of calcium during this period of increased modeling and skeletal consolidation (8,9). The adolescent years are therefore a window of opportunity to influence life-long bone health. Adolescent girls who fail to attain their full growth potential and are malnourished are at risk of having low birth weight babies and obstetric complications (10,11). To significantly reduce the proportion of low-birth-weight babies, the nutritional state of adolescent girls needs urgent attention; hence the need for adolescence girls to achieve the best nutritional status during adolescence before they enter the reproductive period of life (10,11). There is paucity of evidence in Nigeria with regards to the prevalence of hypocalcaemia and its predictors among rural adolescent girls. Therefore, this study was conducted to assess the prevalence and predictors of hypocalcaemia among adolescent girls in rural public secondary schools in south-south Nigeria. This was with the view to providing evidence to inform policy decision making by the relevant authorities concern with adolescent health in public schools in Nigeria.

## Methods

**Study setting**: This study was conducted from September 2017 to January 2018 at nine randomly selected public rural secondary schools in Ethiope West and Ethiope East local government areas (LGAs) of Delta State, south-south Nigeria.

**Study design**: This was a cross-sectional study conducted to assess the prevalence of hypocalcaemia and its predictors among rural school adolescent girls.

**Study population**: The study population comprised of apparently healthy adolescent girls (10 to 19 years) in public secondary schools in Ethiope West and East Local Government Areas of Delta State, south-south Nigeria. The girls were screened before their inclusion in the study. Girls with abnormal clinical findings on clinical evaluation, such as physical and skeletal abnormalities, proven hemoglobinopathy (haemoglobin SS), hemoglobin levels of < 10 g/dl, and abnormal urinary findings on dip stick evaluation using multi-stick medi-test Combi 9 were excluded from the study.

**Sample size calculation**: The minimum sample size was determined based on the prevalence of hypocalcaemia of 6.5% reported among adolescent girls from a previous study (11), an error margin of 5 % and standard normal variate at 95% confidence level. The determined minimum sample size was 100; however, 238 adolescent girls participated in the study.

**Sampling technique**: A multi-stage sampling technique (three stages) was applied in this study. In the first stage, nine public secondary schools (5 from Ethiope West LGA and 4 from Ethiope East LGA) were selected using simple random sampling technique (table of random numbers) from the list of thirty public secondary schools (18 from Ethiope West LGA and 12 from Ethiope East LGA). In the second stage, adolescent girls in the nine selected public secondary schools (Udurhie Sec. School - 150; Ogharaki Model Sec. School - 120; Oginni Sec. School - 260; Oreki Sec. School - 305; Mosogar Sec. School - 127; Erho Sec. School - 164; Oria Sec. School - 280; Abraka Grammer School - 305; and Ovu Grammer School - 300) were proportionately allocated into different strata by their class levels (Junior secondary 2 and 3; senior secondary 1, 2, and 3) to allow for adequate representation; and in the third stage a simple random sampling technique was used to select the study participants, who were randomly selected (table of random numbers) from the school class registers of female students in each class stratum in the selected schools. Female students in junior secondary one (JS1) were excluded from the study. This was because they were just starting their secondary education in the selected schools at the time of data collection and authors had no prior parental consent to draw blood samples from them.

**Study instrument and data collection**: Data was collected using a pre-tested semi-structured questionnaire which was interviewer-administered. A pilot survey was conducted in a public secondary school not selected for the study. The questionnaire was tested for its reliability and was validated with a reliability coefficient of 0.8. The questionnaire elicited information on the socio-demographic characteristics, dietary history, anthropometric indices, and the serum calcium status of the participants. Data was collected from the participants between 10:00 am and 2:00 pm in the selected school premises on the day of data collection. Each participant was interviewed using the study instrument. In addition, anthropometric and serum calcium measurements were carried out.

**Anthropometric measurement**: The weights were measured with the participants standing bare foot and wearing light clothing using a weighing scale. The weight was measured using a weighing scale (Harson Emperor Model H89RED) which had an accuracy of 0.5 kg. The scale was checked for precision each morning using known weights and the necessary adjustments made for recalibration after use for every 50 students.

Height measurements were performed using a portable field wooden stadiometer calibrated to the nearest 0.5 cm. Using a steel measuring tape running from the fixed position at the foot piece to a higher point that was demarcated by the mobile wooden head piece placed at the crown of the head, the participant stood on a flat plane provided by a standing board (Foot Piece) and a mobile head piece was moved to slide until it touches the apex of the head. The height measurement was taken with the subject standing straight on this flat board with the knees together and heels, buttocks and head touching the wall, and looking head on with the chin held horizontal. This was achieved with the help of the research assistant who ensured that the chin was upright in the horizontal plane and the auditory meatus and lower boarder of the orbit in the same horizontal plane; the researcher then marked off the position of the head piece and then read off the measurement from the steel tape.

All weights and height measurements were in triplicate and the mean for each measurement was taken and recorded. The body mass index was calculated from the measured weights and heights of the participants. According to the 2007 BMI-for-age reference for children aged 0–19 years, the cut off values for underweight, normal weight and overweight were taken as BMI < 3^rd^ percentile, BMI between 3^rd^ and 97^th^ percentiles, and BMI > 97^th^ percentile respectively (12).

**Serum calcium measurement**: Two point five millimeters of blood was drawn from each participant for serum calcium ions estimation. The blood samples were drawn from the antecubital vein or dorsal vein of the hand of the participants using a disposable syringes and needles under aseptic conditions without tourniquet. These samples were transferred from the syringe into labeled lithium heparin bottles and transported in cold box with ice packs to the laboratory within 2 hours after collection. The serum calcium ions estimation was done at the research laboratory of the Department of Chemical Pathology of the University of Benin Teaching Hospital by a consultant Chemical pathologist. The ion-selective electrode machine, a genius green medical instrument with electrolyte analyzer model GE200 was used for the serum calcium ions analysis. Serum ionised calcium (free calcium) was analysed because its assessment is a more useful index and provides the better indication of calcium status than total calcium level which is greatly influenced by protein concentration especially albumin (13). The cut off value for normocalcaemia was taken as serum ionized calcium 1.10 - 1.40 mmol/l, while the cut off value for hypocalcaemia was taken as serum ionized calcium < 1.10 mmol/l.

**Outcome and independent variables:** The outcome variable was the prevalence of hypocalcaemia among the study participants. The independent variables include age, class level, social class status, frequency of milk consumption, meal frequently skipped and BMI status. Socioeconomic status was determined using the father's occupation and mother's educational status according to the scoring system described by Olusanya et al (14).

**Statistical analyses**: Data generated was analysed using the IBM SPSS version 22 software. Descriptive and inferential analysis of data collected was carried out. Bivariate analysis using chi-square was carried out and statistical significance set at p < 0.05. To measure the independent effect of different variables on the occurrence of hypocalcaemia, variables that were significant at p-value < 0.05 during the bivariate analysis (Chi-Square analysis) were selected and entered stepwise into the binary logistic regression model. Binary regression analysis was used to obtain the adjusted odds ratio for all variables significant at a p-value < 0.05 during bivariate analysis.

**Ethical considerations**: Ethical clearance was obtained from the research ethics committee of the Delta State University Teaching Hospital, Oghara. Permission was also obtained from the Delta State Ministry of Education and the selected school authorities. A written statement of informed consent was obtained from the parents or guardians of the study participants. The participants were informed of the purpose of the research as well as their right to participate or refuse to participate in the study.

## Results

Over half (53.3%) and three-quarter (75.2%) of the study participants were in their late adolescence period (17–19 years) and belonged to the lower social class level (Table 1). Over one-quarter (30.7%) of the study participants had hypocalcaemia (Table 2).

**Table 1:** Socio-demographic characteristics of the study participants

Variables	Categories	Frequency (%) N=238
**Age (years)**	10–13 (Early adolescence)	23 (9.7)
	14–16 (Mid adolescence)	88 (37.0)
	17–19 (Late adolescence)	127 (53.3)

**Class level**	Junior secondary 2	14 (5.9)
	Junior secondary 3	29 (12.2)
	Senior secondary 1	23 (9.7)
	Senior secondary 2	76 (31.9)
	Senior secondary 3	96 (40.3)

**Social class status**	Middle (social class II)	59 (24.8)
	Lower (social class IV and V)	179 (75.2)

**Table 2:** Serum calcium and body mass index status of the study participants

Variables	Categories	Frequency (%) N=238
**Serum calcium**	Normocalcaemia	165 (69.3)
	Hypocalcaemia	73 (30.7)
**Body mass index**	Normal weight	125 (52.5)
	Underweight	113 (47.5)

The bivariate analysis revealed that the association of the study participants' age (X^2^= 6.51; df = 2; p = 0.04), class level (X^2^= 11.89; df = 4; p = 0.02), frequency of milk consumption (X^2^= 8.45; df = 3; p = 0.02), meal frequently skipped (X^2^= 8.83; df = 2; p = 0.01), and body mass index (X^2^= 5.51; df = 1; p = 0.02) with hypocalcaemia were significant (p < 0.05) (Table 3).

**Table 3:** Predictors of hypocalcaemia among study participants

Variables	Categories	Hypocalcaemia		Bivariate Analysis p value	Regression Analysis AOR (95% C.I.)

		Yes (%) n=73 (30.7)	No (%) n=165 (69.3)		
**Age (years)** **N=238**	10–13	5 (21.7)	18 (78.3)	**0.04**	2.19 (0.76–6.27)
	14–16	20 (22.7)	68 (77.3)		2.38 (1.23–4.57)
	17–19	48 (37.8)	79 (62.2)		1
**Class level** **N=238**	Junior secondary 2	4 (28.6)	10 (71.4)	**0.02**	0.74 (0.15–3.69)
	Junior secondary 3	11 (37.9)	18 (62.1)		0.81(0.26–2.54)
	Senior secondary 1	3 (13.0)	20 (87.0)		4.76 (1.21–18.75)
	Senior secondary 2	16 (21.1)	60 (78.9)		1.98 (0.95–4.13)
	Senior secondary 3	39 (40.6)	57 (59.4)		1
**Social class** **status** **N=238**	Middle	14 (23.7)	45 (76.3)	**0.18**	-
	Lower	59 (33.0)	120(67.0)		
**Frequency of** **milk** **consumption** **N=238**	Never	10 (47.6)	11 (52.4)	**0.04**	1
	Daily	22 (26.5)	61 (73.5)		0.19 (0.06–0.62)
	Weekly	32 (37.2)	54 (62.8)		0.40 (0.16–0.96)
	Monthly	9 (18.7)	39 (81.3)		0.60 (0.24–1.49)
**Meal** **frequently** **skipped** **N=238**	Breakfast	24 (24.2)	75 (75.8)	**0.01**	3.60 (1.65–7.83)
	Lunch	26 (28.6)	65 (71.4)		2.92 (1.35–6.34)
	Dinner	23 (47.9)	25 (52.1)		1
**BMI (Kg/m^2^)** **N=238**	Normal weight	30 (24.0)	95 (76.0)	**0.02**	0.28 (0.19–0.41)
	Underweight	43 (38.1)	70 (61.9)		1

The multivariable analysis revealed that the study participants who were in their mid-adolescence period (14–16 years) (OR= 2.38; 95% CI: 1.23–4.57), who skipped lunch (OR= 2.92; 95% CI: 1.35–6.34), who skipped breakfast (OR= 3.60; 95% CI: 1.65–7.83) and were in senior secondary 1 class (OR= 4.76; 95% CI: 1.21–18.75) had 2, 3, 4, and 5 times higher likelihood respectively of having hypocalcaemia (Table 2). Study participants who consume milk daily (OR= 0.19; 95% CI: 0.06–0.62), who consume milk weekly (OR= 0.40; 95% CI: 0.16–0.96) and who had normal weight (OR= 0.28; 95% CI: 0.19–0.41) had 81.0%, 60.0% and 72.0% decreased odds respectively of having hypocalcaemia (Table 3).

## Discussion

Calcium deficiency and factors that interfere with calcium uptake are critical during the period of adolescence. The prevalence of hypocalcaemia observed among the participants in this study was unacceptably high. This implies a poor nutritional state of the participants in this study and a worrisome development which calls for urgent intervention of all stakeholders involved in adolescent health in the study setting. This is important because unhealthy adolescent girls are likely to become unhealthy mothers who will in turn have unhealthy babies. Therefore, the need for adolescence girls to achieve the best nutritional status during adolescence before they enter the reproductive period of life cannot be overemphasized.

Over one-quarter of the participants in this study were hypocalcaemic compared to less than one-tenth of adolescent girls observed to have hypocalcaemia reported from previous studies conducted in South-east Nigeria (11) and in Tehran, Iran (15) respectively. The marked difference in the prevalence of hypocalcaemia in this study compared to the prevalence reported by the previous studies may not be unconnected with the commonly consumed foods in the study settings. The occurrence of hypocalcaemia in female adolescents has been linked to the consumption of foods like cereals and legumes which have low calcium contents, and most times contain anti-nutritional factors (11). Cereals, legumes and vegetables are known to have low concentration of calcium and need to be commercially fortified to increase their calcium contents. Also, some vegetables contain phytates that interact with calcium metabolism (11). Milk and milk products are rich sources of calcium and calcium in milk is particularly well absorbed than that in plant foods (9). The study participants who consumed milk daily and weekly respectively had decreased odds of becoming hypocalcaemic compared to those who were either not taking milk as part of their diet or taking milk only monthly. This observation is corroborated by the findings from previous studies which have linked poor milk intake to the occurrence of hypocalcaemia (11,16). This is not surprising as the common diets in the study setting are mainly carbohydrate-based consisting of garri, starch, yam, rice, and plantain.

Meal skipping was observed to be significantly associated with the occurrence of hypocalcaemia among the study participants. This observation is corroborated by the findings from a previous study conducted in Nnewi, South-east Nigeria (11). Evidence has shown that food consumption pattern affects an individual's well-being and the choice of which food to eat, where to eat and when to eat are intensely personal and influenced by several factors which in turn influence an individual's nutrient needs (17). The study participants who skipped breakfast and lunch frequently had fourand three-fold odds respectively of becoming hypocalcaemic compared to those who skipped their dinner frequently.

Body mass index was observed to be significantly associated with the occurrence of hypocalcaemia among the study participants. Over three-quarter and three-fifth of the study participants who were normal weight and underweight respectively were hypocalcaemic. However, study participants who were normal weight had decreased odds of becoming hypocalcaemic compared to those who were underweight. This observation is corroborated by the findings from previous studies which have revealed the association of calcium and other micronutrient deficiency states with underweight (16,18,19).

In conclusion, this study brings to the fore a high prevalence of hypocalcaemia among the study participants. The prevention of hypocalcaemia among adolescent girls should be given priority in public secondary schools in rural settings. There is a need to implement health education interventions targeted at parents that emphasize importance of calcium and encourage them to support adolescent girls to take calcium-supplements as well as calcium-rich meals.
